# A prognostic mRNA expression signature of four 16q24.3 genes in radio(chemo)therapy‐treated head and neck squamous cell carcinoma (HNSCC)

**DOI:** 10.1002/1878-0261.12388

**Published:** 2018-10-26

**Authors:** Ludmila Wintergerst, Martin Selmansberger, Cornelius Maihoefer, Lars Schüttrumpf, Axel Walch, Christina Wilke, Adriana Pitea, Christine Woischke, Philipp Baumeister, Thomas Kirchner, Claus Belka, Ute Ganswindt, Horst Zitzelsberger, Kristian Unger, Julia Hess

**Affiliations:** ^1^ Research Unit Radiation Cytogenetics Helmholtz Zentrum München German Research Center for Environmental Health GmbH Neuherberg Germany; ^2^ Clinical Cooperation Group ‘Personalized Radiotherapy in Head and Neck Cancer’ Helmholtz Zentrum München German Research Center for Environmental Health GmbH Neuherberg Germany; ^3^ Department of Radiation Oncology University Hospital LMU Munich Germany; ^4^ Research Unit Analytical Pathology Helmholtz Zentrum München German Research Center for Environmental Health GmbH Neuherberg Germany; ^5^ Institute of Computational Biology Helmholtz Zentrum München German Research Center for Environmental Health GmbH Neuherberg Germany; ^6^ Institute of Pathology Faculty of Medicine LMU Munich Germany; ^7^ Department of Otorhinolaryngology, Head and Neck Surgery University Hospital LMU Munich Germany; ^8^ German Cancer Consortium (DKTK) Munich Germany; ^9^ Department of Therapeutic Radiology and Oncology Innsbruck Medical University Austria

**Keywords:** classifier, gene association network, head and neck squamous cell carcinoma, prognosis, radiotherapy, signature

## Abstract

Previously, we have shown that copy number gain of the chromosomal band 16q24.3 is associated with impaired clinical outcome of radiotherapy‐treated head and neck squamous cell carcinoma (HNSCC) patients. We set out to identify a prognostic mRNA signature from genes located on 16q24.3 in radio(chemo)therapy‐treated HNSCC patients of the TCGA (The Cancer Genome Atlas, *n* = 99) cohort. We applied stepwise forward selection using expression data of 41 16q24.3 genes. The resulting optimal Cox‐proportional hazards regression model included the genes APRT, CENPBD1, CHMP1A, and GALNS. Afterward, the prognostic value of the classifier was confirmed in an independent cohort of HNSCC patients treated by adjuvant radio(chemo)therapy (LMU‐KKG cohort). The signature significantly differentiated high‐ and low‐risk patients with regard to overall survival (HR = 2.01, 95% CI 1.10–3.70; *P* = 0.02125), recurrence‐free survival (HR = 1.84, 95% CI 1.01–3.34; *P* = 0.04206), and locoregional recurrence‐free survival (HR = 1.87, 95% CI 1.03–3.40; *P* = 0.03641). The functional impact of the four signature genes was investigated after reconstruction of a gene association network from transcriptome data of the TCGA HNSCC cohort using a partial correlation approach. Subsequent pathway enrichment analysis of the network neighborhood (first and second) of the signature genes suggests involvement of HNSCC‐associated signaling pathways such as apoptosis, cell cycle, cell adhesion, EGFR, JAK‐STAT, and mTOR. Furthermore, a detailed analysis of the first neighborhood revealed a cluster of co‐expressed genes located on chromosome 16q, substantiating the impact of 16q24.3 alterations in poor clinical outcome of HNSCC. The reported gene expression signature represents a prognostic marker in HNSCC patients following postoperative radio(chemo)therapy.

**Abbreviations**
API: Application Programming Interface
APRT: adenine phosphoribosyltransferase
CENPBD1: CENPB DNA‐binding domain containing 1
CHMP1A: charged multivesicular body protein 1A
CI: confidence interval
FDR: false discovery rate
FFPE: formalin‐fixed paraffin‐embedded
GALNS: galactosamine (N‐acetyl)‐6‐sulfatase
GAN: gene association network
GSEA: gene set enrichment analysis
HE: hemotoxylin and eosin
HNSCC: head and neck squamous cell carcinoma
HPV: human papilloma virus
HR: hazard ratio
IHC: immunohistochemistry
NE: not estimable
OS: overall survival
PGK1: phosphoglycerate kinase 1
q‐PCR: quantitative real‐time polymerase chain reaction
qRT‐PCR: quantitative real‐time reverse transcription polymerase chain reaction
SNP: single nucleotide polymorphisms
TPM: Transcripts Per Million

## 1 Introduction

Head and neck squamous cell carcinoma (HNSCC) comprises a group of heterogeneous tumors from different anatomical sites with tobacco smoking and alcohol abuse as the major risk factors (Marcu and Yeoh, 2009). Infection with high‐risk human papillomavirus (HPV) is another emerging risk factor. HPV‐related tumors are characterized by a distinct molecular pathogenesis with a considerably favorable prognosis (Leemans *et al*., 2011; O'Sullivan *et al*., 2013). Despite advances in treatment modalities, the 5‐year survival rate for advanced HNSCC still needs improvement. The identification of patients with therapy‐resistant tumors using prognostic markers would allow personalized treatment approaches (Mirghani *et al*., 2015; O'Sullivan *et al*., 2013); however, such markers are not yet established.

So far, research groups have mainly focused on the identification of single molecular markers to be used for diagnosis and treatment selection in HNSCC (Rocco *et al*., 2006). Due to the complexity and heterogeneity of tumors, it is likely that instead of a single gene, the prognostic value of a gene signature with regard to patient outcome would be more powerful (Ginos *et al*., 2004; Lohavanichbutr *et al*., 2013; Pavon *et al*., 2012). Up to now, mRNA expression signatures associated with metastasis (Lian *et al*., 2013; Roepman *et al*., 2006), hypoxia (Eustace *et al*., 2013; Toustrup *et al*., 2012), HPV status (Cancer Genome Atlas, 2015; Slebos *et al*., 2006), and immune response (Chung *et al*., 2004; Wood *et al*., 2016) have been reported in HNSCC. Although, for example, the immune response signature of Chung *et al*. and the hypoxia signatures developed by Eustace *et al*. and Toustrup *et al*. could be confirmed in subsequent publications (Keck *et al*., 2015; Tawk *et al*., 2016), many molecular signatures, some of them very complex, fail independent validation and therefore to change practice in a clinical setting. This might be explained by methodological aspects such as the selection and number of genes examined, differences in the analysis platforms used, restrictions due to small sample sizes, lack of independent validation, but also by demographic differences in the patient groups examined or the unavailability of detailed clinical information.

Previously, we demonstrated an association of gains of chromosomal band 16q24.3 with locoregional progression‐free survival of radiotherapy‐treated HNSCC patients (Bauer *et al*., 2008) and validated this marker in the subgroup of adjuvant radio(chemo)therapy‐treated patients of the TCGA HNSCC cohort (Hess *et al*., 2017). Since copy number alterations might lead to aberrant mRNA expression of genes (Gollin, 2014), our objective was to develop a prognostic mRNA signature from genes located on this chromosomal band using a stepwise forward selection approach. The prognostic value of the gene classifier was analyzed in an independent HNSCC cohort. In addition, the functional role of the signature genes was investigated.

## 2 Methods

### 2.1 Patients

Our study was conducted in compliance with the Reporting recommendations for tumor MARKer prognostic studies (REMARK) (McShane *et al*., 2005) and fulfills the requirements set out by Simon *et al*. (2009), which are summarized in Table S1 (Simon *et al*., 2009).

We analyzed two independent cohorts of HNSCC patients who had undergone surgical resection followed by adjuvant radio(chemo)therapy: the TCGA and the LMU‐KKG cohort (Ludwig‐Maximilians‐University of Munich, Clinical Cooperation Group ‘Personalized Radiotherapy in Head and Neck Cancer’) (Maihoefer *et al*., 2018).

For the TCGA HNSCC cohort, mRNA expression (RNA Seq V2 RSEM)‐level *z*‐scores of genes located on chromosomal band 16q24.3 (generated by the TCGA Research Network http://cancergenome.nih.gov) were downloaded from cBioPortal (2015/08/12) (Cancer Genome Atlas, 2015; Cerami *et al*., 2012; Gao *et al*., 2013). Inclusion criteria for patients of the TCGA cohort were as follows: (a) Treatment with radio(chemo)therapy; (b) no therapy in the frame of neoadjuvant, recurrent, or palliative treatment; (c) availability of HPV status; (d) availability of mRNA expression and genomic copy number data of genes located on chromosomal band 16q24.3. The resulting patient subset of 99 radio(chemo)therapy‐treated HNSCC patients (Table S2) was randomly split into a training (*n* = 40) and a validation set (*n* = 59) while ensuring equal percentage distribution of HPV‐positive cases. Median follow‐up times of the training and validation set were 656 and 643 days, respectively.

The retrospective LMU‐KKG cohort served as an independent validation cohort. This study on clinical and biological data was approved by the local ethics committee in Munich (EA 448‐13 and 17‐116) and carried out in accordance with the Declaration of Helsinki. The experiments were undertaken with the understanding and written consent of each subject. The LMU‐KKG cohort included all patients with HNSCC of the hypopharynx, larynx, oropharynx, or oral cavity treated at the Department of Radiation Oncology, LMU, Germany, between 2008 and 2013 (Maihoefer *et al*., 2018). All patients received adjuvant radiotherapy as a curative approach after surgical resection. The median overall treatment time was 45 days (interquartile range 43–47 days) with five fractions per week. A median radiation dose of 64 Gy (2 Gy/fraction) was applied to the former tumor bed or regions of ECE. Elective lymph node regions have been covered according to tumor stage and localization with a median dose of 50 Gy (2 Gy/fraction) and 56 Gy (2 Gy/fraction) were applied to involved lymph node regions. In the case of close (R0, but less than 5 mm) or positive microscopic resection margins and/or ECE, patients received concurrent chemotherapy; 47.2% of the patients received CDDP/5‐fluorouracil (5‐FU) (CDDP: 20 mg·m^−2^ days 1–5/29–33; 5‐FU: 600 mg·m^−2^ days 1–5/29–33). In some cases, Mitomycin C (MMC) (10.2%) or 5‐FU/MMC (5.6%) was used instead of platinum‐based chemotherapy. End of follow‐up period was on 14 May 2016, and the median OS time was 1878 days. The clinical and pathological data for both cohorts are presented in Table 1. A comparison of demographic parameters between the TCGA and the LMU‐KKG cohort is given in Table S3.

**Table 1:** Demographic and treatment characteristics of the total adjuvant radio(chemo)therapy‐treated TCGA HNSCC cohort, of the TCGA HNSCC training and validation set and the adjuvant radio(chemo)therapy‐treated LMU‐KKG HNSCC patient cohort and stratified according to the four‐gene signature. % represents percentage of all patients

	TCGA cohort all (*n* = 99)	TCGA training set (*n* = 40)				TCGA validation set (*n* = 59)				LMU‐KKG cohort (*n* = 108)			
	No. of all patients	No. of all patients	Low‐risk (*n* = 24)	High‐risk (*n* = 16)	*P*‐value^a^	No. of all patients	Low‐risk (*n* = 30)	High‐risk (*n* = 29)	*P*‐value^a^	No. of all patients	Low‐risk (*n* = 53)	High‐risk (*n* = 55)	*P*‐value^a^
Sex, no. (%)													
Male	79 (80)	35 (88)	22 (92)	13 (81)	0.37	44 (75)	24 (80)	20 (69)	0.38	74 (69)	37 (70)	37 (67)	0.84
Female	20 (20)	5 (13)	2 (8)	3 (19)		15 (25)	6 (20)	9 (31)		34 (31)	16 (30)	18 (33)	
Age (years), no. (%)													
< 60	56 (57)	23 (58)	12 (50)	11 (69)	0.33	33 (56)	16 (53)	17 (59)	0.79	44 (41)	25 (47)	19 (35)	0.24
≥ 60	43 (43)	17 (43)	12 (50)	5 (31)		26 (44)	14 (47)	12 (41)		64 (59)	28 (53)	36 (65)	
Tumor localization, no. (%)													
Oral cavity	49 (49)	17 (43)	8 (33)	9 (56)	0.067	32 (54)	14 (47)	18 (62)	0.51	27 (25)	14 (26)	13 (24)	0.91
Larynx	28 (28)	14 (35)	8 (33)	6 (38)		14 (24)	8 (27)	6 (21)		12 (11)	6 (11)	6 (11)	
Oropharynx	20 (20)	7 (18)	7 (29)	0 (0)		13 (22)	8 (27)	5 (17)		52 (48)	26 (49)	26 (47)	
Hypopharynx	2 (2)	2 (5)	1 (4)	1 (6)		0 (0)	0 (0)	0 (0)		17 (16)	7 (13)	10 (18)	
Alcohol consumption, no. (%)													
Yes	84 (85)	33 (83)	19 (79)	14 (88)	0.38	51 (86)	25 (83)	26 (90)	1	53 (49)	27 (51)	26 (47)	1
No	13 (13)	6 (15)	5 (21)	1 (6)		7 (12)	4 (13)	3 (10)		32 (30)	16 (30)	16 (29)	
Missing information	2 (2)	1 (3)	0 (0)	1 (6)		1 (2)	1 (3)	0 (0)		23 (21)	10 (19)	13 (24)	
Smoking status, no. (%)													
Smoker	80 (81)	30 (75)	16 (67)	14 (88)	0.26	50 (85)	26 (87)	24 (83)	0.73	71 (66)	29 (55)	42 (76)	1
Never smoker	19 (19)	10 (25)	8 (33)	2 (13)		9 (15)	4 (13)	5 (17)		9 (8)	4 (8)	5 (9)	
Missing information	0 (0)	0 (0)	0 (0)	0 (0)		0 (0)	0 (0)	0 (0)		28 (26)	20 (38)	8 (15)	
UICC TNM stage, no. (%)													
I	2 (2)	0 (0)	0 (0)	0 (0)	0.84	2 (3)	2 (7)	0 (0)	0.089	1 (1)	0 (0)	1 (2)	0.92
II	4 (4)	3 (8)	2 (8)	1 (6)		1 (2)	1 (3)	0 (0)		10 (9)	5 (9)	5 (9)	
III	7 (7)	1 (3)	1 (4)	0 (0)		6 (10)	1 (3)	5 (17)		33 (31)	18 (34)	15 (27)	
IVa	57 (58)	23 (58)	14 (58)	9 (56)		34 (58)	18 (60)	16 (55)		61 (56)	29 (55)	32 (58)	
IVb	1 (1)	1 (3)	0 (0)	1 (6)		0 (0)	0 (0)	0 (0)		3 (3)	1 (2)	2 (4)	
Missing information	28 (28)	12 (30)	7 (29)	5 (31)		16 (27)	8 (27)	8 (28)		0 (0)	0 (0)	0 (0)	
pT stage, no. (%)													
T1	5 (5)	1 (3)	1 (4)	0 (0)	1	4 (7)	2 (7)	2 (7)	0.59	19 (18)	10 (19)	9 (16)	0.31
T2	18 (18)	9 (23)	6 (25)	3 (19)		9 (15)	4 (13)	5 (17)		45 (42)	18 (34)	27 (49)	
T3	13 (13)	3 (8)	2 (8)	1 (6)		10 (17)	7 (23)	3 (10)		29 (27)	18 (34)	11 (20)	
T4	37 (7)	17 (43)	10 (42)	7 (44)		20 (34)	9 (30)	11 (38)		15 (14)	7 (13)	8 (15)	
Missing information	26 (26)	10 (25)	5 (21)	5 (31)		16 (27)	8 (27)	8 (28)		0 (0)	0 (0)	0 (0)	
pN stage, no. (%)													
N0	20 (20)	9 (23)	4 (17)	5 (31)	0.63	11 (19)	5 (17)	6 (21)	1	31 (29)	16 (30)	15 (27)	0.95
N1	11 (11)	3 (8)	2 (8)	1 (6)		8 (14)	4 (13)	4 (14)		26 (24)	7 (13)	13 (24)	
N2	39 (39)	16 (40)	11 (46)	5 (31)		23 (39)	12 (40)	11 (38)		49 (45)	23 (43)	26 (47)	
N3	0 (0)	0 (0)	0 (0)	0 (0)		0 (0)	0 (0)	0 (0)		2 (2)	1 (2)	1 (2)	
Missing information	29 (29)	12 (30)	7 (29)	5 (31)		17 (29)	9 (30)	8 (28)		0 (0)	0 (0)	0 (0)	
Grade, no. (%)													
G1 (well differentiated)	5 (5)	0 (0)	0 (0)	0 (0)	0.74	5 (8)	3 (10)	2 (7)	0.64	3 (3)	3 (6)	0 (0)	0.11
G2 (moderate differentiated)	62 (63)	25 (63)	14 (58)	11 (69)		37 (63)	16 (53)	21 (72)		37 (34)	15 (28)	22 (40)	
G3 (poorly differentiated)	29 (29)	15 (38)	10 (42)	5 (31)		14 (24)	8 (27)	6 (21)		68 (63)	35 (66)	33 (60)	
Missing information	3 (3)	0 (0)	0 (0)	0 (0)		3 (5)	3 (10)	0 (0)		0 (0)	0 (0)	0 (0)	
HPV status, no. (%)													
Positive	21 (21)	9 (23)	9 (38)	0 (0)	0.006	12 (20)	11 (37)	1 (3)	0.00247	23 (21)	10 (19)	13 (24)	0.64
Negative	78 (79)	31 (78)	15 (62)	16 (100)		47 (80)	19 (63)	28 (97)		85 (79)	43 (81)	42 (76)	
Simultaneous chemotherapy, no. (%)													
Platinum‐based	55 (56)	22 (55)	15 (63)	7 (44)	0.29	33 (56)	18 (60)	15 (52)	0.15	54 (50)	28 (53)	26 (47)	0.71
Other	4 (4)	1 (3)	0 (0)	1 (6)		3 (5)	3 (10)	0 (0)		18 (17)	7 (13)	11 (20)	
No	26 (26)	8 (20)	4 (17)	4 (25)		18 (31)	7 (23)	11 (38)		36 (33)	18 (34)	18 (33)	
Missing information	14 (14)	9 (23)	5 (21)	4 (25)		5 (8)	2 (7)	3 (10)		0 (0)	0 (0)	0 (0)	

a Chi‐square test or Fisher's exact test.

Hemotoxylin and eosin (HE)‐stained tissue sections from available formalin‐fixed and paraffin‐embedded (FFPE) tissue blocks were histopathologically reviewed by a pathologist (A.W.), and the tumor containing area was defined. Samples with < 50% tumor cells were excluded from further analysis. Guided by the HE‐stained tissue slides, the annotated tumor area was micro‐dissected followed by simultaneous DNA and RNA extraction using the AllPrep DNA/RNA FFPE Kit (Qiagen, Hilden, Germany) according to the manufacturer's protocol. After DNA/RNA isolation and quality assessment, 108 HNSCC samples remained for further analysis.

### 2.2 Determination of the HPV status

HPV status of the patients was determined by p16^INK4a^ immunohistochemistry in combination with HPV DNA detection. Immunohistochemical (IHC) p16^INK4a^ staining, used as a surrogate marker for HPV‐infection, was performed using the CINtec TM Histology Kit (Roche mtm Laboratories AG, Heidelberg, Germany) on a Ventana Benchmark LT automated immunostainer (Ventana Medical Systems, Tucson, AZ, USA) according to the manufacturer's protocol. FFPE sections of embedded p16‐positive (UPCI SCC154) and p16‐negative HNSCC cell lines (Cal33) were included as positive and negative controls. Tumor specimens with strong and diffuse nuclear and cytoplasmic staining in more than 70% of tumor cells were considered as p16‐positive, whereas tissues with only faintly diffuse or absent staining were considered as p16‐negative (Ang *et al*., 2010). p16‐stained tissue sections were evaluated by two independent observers (L.W. and J.H.). Further, detection of mucosotropic HPV DNA was performed using quantitative real‐time PCR (q‐PCR) in combination with SYBR green chemistry (Clontech Laboratories, Inc., Mountain View, CA, USA). DNA samples (50 ng) were subjected to q‐PCR products (10 μL) on a ViiA 7 q‐PCR system (Thermo Fisher Scientific, Dreieich, Germany) using GP5+/6+ primers detecting the L1 gene (Eurofins MWG Operon, Ebersberg, Germany) (forward primer: 5′‐TTTGTTACTGTGGTAGATACTAC‐3′, reverse primer: 5′‐GAAAAATAAACTGTAAATCATATTC‐3′; amplicon size: 142 bp) (Hesselink *et al*., 2005). The β‐globin gene served as quality control (forward primer: 5′‐CAGGTACGGCTGTCATCACTTAGA‐3′, reverse primer: 5′‐CATGGTGTCTGTTTGAGGTTGCTA‐3′; amplicon size: 185 bp) *(*Metabion International AG, Planegg‐Martinsried, Germany) (Lindh *et al*., 2007). Two HPV‐positive (UPCI SCC2 and UPCI SCC154) and two HPV‐negative (Cal27 and Cal33) cell lines were included as controls. Reactions were carried out in triplicates along with negative controls. Samples with a detectable β‐globin PCR product (Ct‐value < 35) were considered as HPV‐negative if no HPV amplification product was detectable.

A tumor specimen was classified as HPV‐positive if it was positive for both p16^INK4a^ IHC and HPV DNA status assessed by GP 5+/6+ q‐PCR (Smeets *et al*., 2007).

### 2.3 Development of a gene classifier

A schematic workflow for the development of the gene classifier and the reconstruction of the gene association network (GAN) with subsequent analyses is presented in Fig. S1.

A prognostic gene classifier regarding OS was built by applying a robust likelihood‐based survival modeling approach on mRNA gene expression data (*z*‐scores) of the TCGA training set using the R‐package rbsurv (Cho *et al*., 2009). A stepwise forward selection algorithm computed the partial likelihood of the Cox‐proportional hazards regression model for a sequential selection of mRNA (100 iterations, twofold cross‐validation), which allowed choosing the best performing model based on the Akaike information criterion, an estimator of the relative quality of a statistical model considering the goodness of fit penalized by model complexity, in terms of the best trade‐off between minimum complexity and best goodness of fit of the model.

Cox model coefficients [adenine phosphoribosyltransferase (APRT): 1.20988822, CENPBD1: 0.06012163, CHMP1A: 0.17153750, GALNS: 0.22431583] were multiplied with the corresponding mRNA expression values and summed up to an individual risk score for each patient. For the determination of the cutoff for the individual risk score stratifier, survival analyses were performed in the TCGA training set with cutoff values varying with increment of 0.1 starting from the minimal risk score. The cutoff, which resulted in the optimal split of patients in terms of log‐rank test *P*‐value (OS) in the training set, was used for assorting each patient individually into a low‐risk (< −0.2932616) and a high‐risk group (≥ −0.2932616). Both the model and the cutoff value derived from the TCGA training dataset were applied to the expression data of the TCGA validation set and the LMU‐KKG cohort. Expression data for the LMU‐KKG cohort were gained from quantitative real‐time RT‐PCR (qRT‐PCR) analysis.

### 2.4 Quantitative real‐time RT‐PCR (qRT‐PCR) analysis of the signature genes in the LMU‐KKG cohort

For the LMU‐KKG HNSCC cohort, mRNA expression of the signature genes was analyzed by qRT‐PCR. Reverse transcription was performed using the SuperScript^®^ VILO™ cDNA Synthesis Kit (Life Technologies, Carlsbad, CA, USA) with 500 ng RNA input. qRT‐PCR products (10 μL) were carried out in triplicates according to manufacturer's protocol on an Applied Biosystems^®^ ViiA™7 platform employing the Taqman^®^ Assays (Life Technologies) APRT‐Hs00975725_m1, CHMP1A‐Hs00946132_g1, CENP BD1‐Hs00924894_s1, and GALNS‐Hs00975732_m1. β‐Actin (ACTB‐Hs01060665_g1) and phosphoglycerate kinase 1 (PGK1‐Hs99999906_m1) served as endogenous controls. Expression levels were determined using the ΔCt method followed by *z*‐score transformation.

### 2.5 Clinical endpoints LMU‐KKG cohort

Clinical endpoints included OS, recurrence‐free survival, and locoregional recurrence‐free survival. OS was calculated (in days) from the date of radiotherapy treatment start to the date of death from any cause. Recurrence‐free survival was defined as the time (days) from the start of radio(chemo)therapy treatment to the first observation of locoregional/distant recurrence or death due to any cause; locoregional recurrence‐free survival from the start of radio(chemo)therapy treatment to the date of local recurrence or death due to any cause. In the absence of an event, patients were censored at the date of the last follow‐up visit.

### 2.6 Genomic copy number data

File IDs of patients of the adjuvant radio(chemo)therapy‐treated TCGA HNSCC cohort were extracted using the GDC web API. Genomic copy number alterations SNP 6.0 raw data (.CEL files) were downloaded from the GDC data portal (https://portal.gdc.cancer.gov/). The CEL files were batch processed using functions of the rawcopy R‐package (http://rawcopy.org). The normal tissue data served as reference data to build normalized log_2_ intensity copy number ratios for each of the tumor samples. The log_2_‐ratios were segmented and the copy number status determined using functions of the R‐package CGHcall (van de Wiel *et al*., 2007). After determination of the DNA status, integration analysis of copy number data with the transcriptome data was carried out.

### 2.7 Statistical analysis

Comparisons of two Kaplan–Meier curves were performed using the log‐rank test of the R‐package survival; *P*‐values < 0.05 were considered statistically significant. Median estimates and hazard ratios (HR) with 95% confidence intervals (CI) were determined. *P*‐values were also calculated for the training set, but since no valid null‐hypothesis can be formulated in this case, it only reflects the meaningful split of risk groups. The association of clinical parameters with clinical endpoints was assessed using univariate Cox‐proportional hazards regression analysis. Parameters with *P* < 0.05 in univariate analysis were included into a multivariate Cox‐proportional hazards model.

Association of the copy number status of chromosomal band 16q24.3 with risk scores or mRNA expression levels of the signature genes was evaluated using unpaired two‐sided Mann–Whitney *U*‐test.

### 2.8 Gene association network (GAN) reconstruction

Raw RNA sequencing data on 98 HNSCC cases of the adjuvant radio(chemo)therapy‐treated TCGA HNSCC cohort were downloaded from the GDC data portal (https://portal.gdc.cancer.gov/). The RNA sequencing reads contained in the BAM files were realigned to the reference transcriptome (GRCH38/Ensembel) and quantified as Transcripts Per Million (TPM) using the tool Salmon (Patro *et al*., 2017). The identification of differentially expressed genes between the high‐risk and low‐risk group was carried out using the R‐package DEseq2*,* where genes with a cumulative (over samples) TPM ≤ 5 were excluded and genes with an adjusted *P*‐value < 0.1 were considered statistically significant (Love *et al*., 2014).

Differentially expressed genes were subsequently subjected to GAN reconstruction using the method implemented in the GeneNet R‐package, which is based on regularized dynamic partial correlation (Opgen‐Rhein and Strimmer, 2007). The edge probability cutoff of 0.99 was applied to obtain the resulting undirected GAN.

### 2.9 Gene set enrichment analysis

Gene sets from the Reactome database for gene set enrichment analysis (GSEA) were downloaded from the Broad Institute Molecular Signatures Database. GSEA was carried out in the preranked mode: All genes of the data set were ranked according to the log_2_ transformed fold changes (four‐gene signature high‐risk versus low‐risk group). GSEA tests for up‐ or down‐regulation of gene sets (pathways), while gene sets with false discovery rate (FDR) < 0.05 were considered statistically significant.

### 2.10 Pathway enrichment analysis

The Cytoscape Reactome Functional Interaction (FI) plugin (version 3.5.1) was used to perform a network clustering of the FI network consisting of genes from the GANs first and second neighborhood of the four signature genes (Shannon *et al*., 2003; Wu *et al*., 2010). A pathway enrichment analysis was conducted for modules including more than 100 genes. Pathways containing less than 200 but more than 20 genes were considered for pathway enrichment analysis. *P*‐values were determined by one‐sided Fisher's exact test. Benjamini–Hochberg adjusted *P*‐values (FDR) < 0.05 were considered statistically significant and ranked according to ascending FDRs.

### 2.11 Integration of copy number data with the transcriptome data

In order to assess whether mRNA expressions were explainable by genomic copy number alterations, an integration analysis was carried out on 98 HNSCC samples from the TCGA cohort. For this purpose, the Gene wise cisTest implemented in the Bioconductor sigaR R‐package was used (van Wieringen and van de Wiel, 2009; van Wieringen *et al*., 2012). Default values provided by the package were applied during the different analysis steps for all parameters, except nGenes = 500, which potentially improves the overall power of the FDR procedure included in the cisEffectTune function. Overall, this integrative analysis assesses the effect of the genomic copy number status of each gene on its mRNA expression level and provides a measure on how much of the mRNA expression is explained by its copy number status. The results are based on a permutation approach, while 10 000 permutations were used. The resulted *P*‐values were adjusted for multiple testing with the Benjamini–Hochberg method. Due to the nature of permutation tests, the results vary upon repetition of the test but stay stable with the respect to the drawn conclusions.

## 3 Results

**Figure 1:**
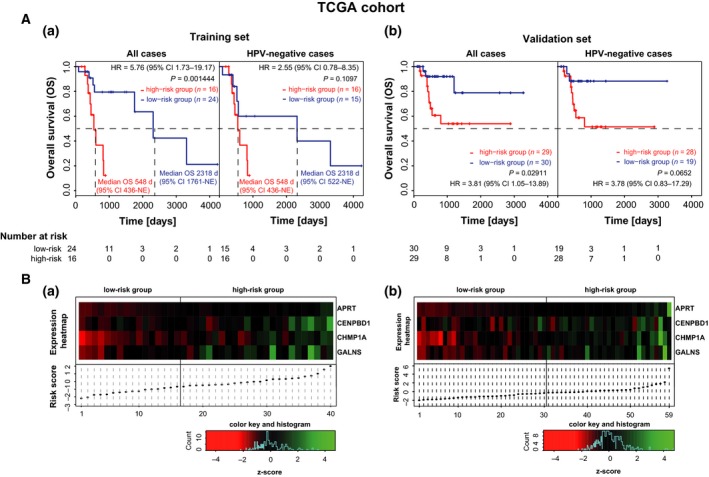
Identification of a four‐gene classifier predicting OS in the subgroup of radio(chemo)therapy‐treated HNSCC patients of the TCGA cohort. (A) Kaplan–Meier curves for the endpoint OS for patients stratified according to the four‐gene classifier of the training (a) and validation (b) set within the radio(chemo)therapy‐treated TCGA HNSCC cohort. Survival curves are depicted for all cases (training set: *n* = 40, validation set: *n* = 59) and for HPV‐negative cases only (training set: *n* = 31, validation set: *n* = 47). *P*‐values, median OS times, and hazard ratios (HR) with 95% contingency intervals were obtained by Log‐rank test and are indicated. (B) Heatmap of mRNA expression levels (*z*‐scores) of the four signature genes (top panel) arranged according to risk scores (bottom panel) for the training (a) and validation (b) set within the TCGA HNSCC cohort. mRNA expression levels of all four signature genes were elevated in patients of the high‐risk group. The results are in whole based upon data generated by the TCGA Research Network: http://cancergenome.nih.gov (Cancer Genome Atlas, 2015; Cerami *et al*., 2012; Gao *et al*., 2013). NE, not estimable.

The identified best performing prognostic model generated from mRNA expression data of 16q24.3 genes (*n* = 41) included the four genes APRT, CENPBD1, CHMP1A, and GALNS allowing to stratify HNSCC patients with regard to OS. A 5.76‐fold (95% CI 1.73–19.17) increased risk for death was observed for high‐risk patients (9/16, 56% patients with events) compared to the low‐risk group (7/24, 29% patients with events) of the TCGA training set (*n* = 40) (Fig. 1A). The robustness of the four‐gene classifier was confirmed in the TCGA validation set as high‐risk classified patients showed significantly reduced OS rates (10/29, 34% patients with events; hazard ratio (HR) 3.81, 95% CI 1.05–13.89; *P* = 0.02911; Fig. 1B) compared to low‐risk patients (3/30, 10% patients with events).

The mRNA expression levels of all signature genes were up‐regulated in high‐risk patients and correlated positively with the defined risk groups (Fig. 1A,B, lower panel).

**Figure 2:**
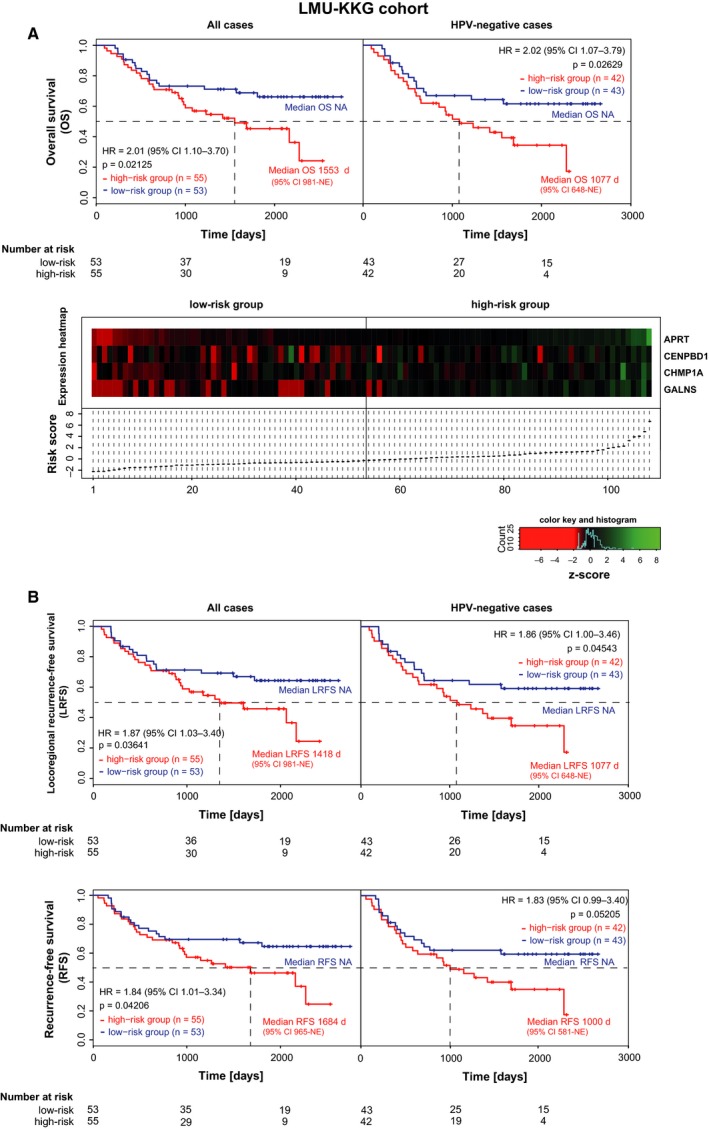
Validation of the extracted four‐gene classifier in an independent HNSCC cohort. Kaplan–Meier curves for the endpoints overall (A), locoregional recurrence‐free and recurrence‐free survival (B) for patients stratified according to the four‐gene classifier of the adjuvant radio(chemo)therapy‐treated LMU‐KKG HNSCC cohort. Survival curves are shown for all cases (left panel) and for the HPV‐negative cases (right panel). Hazard ratios and median survival times with 95% contingency intervals and *P*‐values were calculated by Log‐rank test. Heatmap of mRNA expression levels of the four signature genes arranged according to risk scores (A, lower panel) for patients of the adjuvant radio(chemo)therapy‐treated LMU‐KKG cohort. All genes show a tendency toward higher expression in patients of the high‐risk group. NE, not estimable.

The four‐gene classifier was independently validated in the LMU‐KKG cohort (*n* = 108) as high‐risk group patients showed significantly impaired OS rates (29/55, 45% patients with events; HR 2.01, 95% CI 1.10–3.70; *P* = 0.02125) compared to low‐risk patients (17/53, 32% patients with events) (Fig. 2A). Moreover, the risk groups significantly differed with regard to locoregional recurrence‐free survival (HR = 1.87, 95% CI 1.03–3.40; *P* = 0.03641) and recurrence‐free survival rates (HR = 1.84, 95% CI 1.01–3.34; *P* = 0.04206; Fig. 2B).

In order to assess whether the classifier was an independent prognostic factor, associations of known clinicopathological factors with the high‐ and low‐risk groups were tested. HPV status was associated with the signature‐defined risk groups of the TCGA training and validation set (Table 1). All other demographic parameters were equally distributed among the risk groups in both TCGA subsets and the LMU‐KKG HNSCC cohort. Univariate Cox regression analyses revealed a significant association of HPV status with OS in both cohorts, which was also reflected by an improved OS of oropharyngeal tumors (*n* = 7; all HPV‐positive) compared to tumors of the oral cavity in the TCGA sets (Table S4).

Due to the lack of the presence of HPV‐positive cases in the TCGA high‐risk groups, it was unfeasible to conduct a multivariate analysis. Instead, survival analyses were carried out stratified to HPV‐negative patients demonstrating a trend toward worse survival of high‐risk patients despite small group sizes in the TCGA subsets (Fig. 1). An additional survival analysis performed in the pooled HPV‐negative tumors of the TCGA subsets (training and validation) demonstrated a significant separation of the four‐gene signature classified risk groups low‐risk and high‐risk (HR 2.60, 95% CI 1.08–6.26; *P* = 0.001444) (Fig. S2). Inclusion of HPV status in a multivariate Cox‐proportional hazard model in the LMU‐KKG cohort revealed a significant contribution of the four‐gene classifier and HPV status to the prediction model (Table 2). The inclusion of both parameters HPV status and the four‐gene signature in the model was justified because there was no significant association between them (Fisher's exact test *P*‐value = 0.6406; Table 1). An additional stratified model including an interaction term revealed no significant interaction between the four‐gene signature and HPV status (*P* = 0.73). However, due to the low number of events in the group of HPV‐positive cases (4/23 events; 17% of patients), the results cannot consider being meaningful. Instead, we performed survival analyses considering HPV‐negative cases only (Fig. 2).

**Table 2:** Multivariate Cox regression analysis of the four‐gene classifier and HPV status with OS in the LMU‐KKG HNSCC cohort

Cohort	Parameter	No. of cases	HR	95% CI HR	*P*‐value (univariate analysis)	*P*‐value
LMU‐KKG	Four‐gene classifier (high‐risk vs low‐risk)	55 vs 53	2.17	1.18‐4.00	0.022	0.013
	+					
	HPV status (negative vs positive)	85 vs 23	4.04	1.44‐11.30	0.0067	0.0078

**Figure 3:**
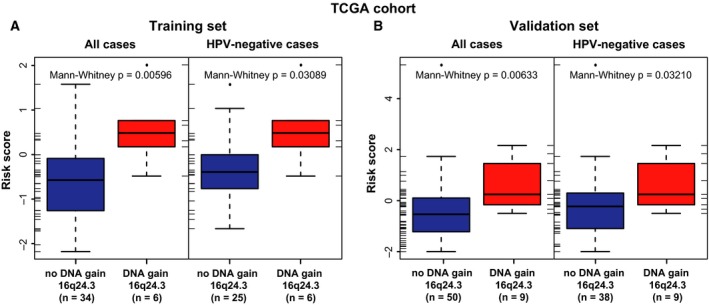
DNA gains of chromosomal band 16q24.3 are associated with increased risk scores in radio(chemo)therapy‐treated patients of the TCGA HNSCC training and validation set. Distribution of risk scores in all (left panel) and in HPV‐negative (right panel) cases with gain and without gain of the chromosomal band 16q24.3 in the training (all cases: *n* = 40, HPV‐negative cases: *n* = 31) (A) and validation (all cases: *n* = 59, HPV‐negative cases: *n* = 47) (B) set within the subgroup of radio(chemo)therapy‐treated HNSCC of the TCGA cohort. Patients with a DNA gain of 16q24.3 had significantly higher risk scores compared to patients without a DNA gain of 16q24.3 (Mann–Whitney *U*‐test). The association remained after stratification to HPV‐negative patients. The results are in whole based upon data generated by the TCGA Research Network: http://cancergenome.nih.gov (Cancer Genome Atlas, 2015; Cerami *et al*., 2012; Gao *et al*., 2013).

Next, we assessed the association of DNA gains of 16q24.3 with mRNA expression levels of the signature genes and the four‐gene classifier risk scores. In both TCGA subsets, significantly higher (*P* < 0.05) risk scores were observed for HNSCC cases with compared to cases without DNA gains of 16q24.3, also when stratified to HPV‐negative patients (Fig. 3). This was also the case on single gene level (Fig. S3).

To get insights into the biological functions of the signature genes, a GSEA of gene expression data with regard to the signature‐defined high‐ versus low‐risk groups was performed. Twenty‐nine significantly (FDR < 0.05) up‐regulated (Table S5) and 53 down‐regulated gene sets (Table S6), respectively, in the high‐risk group compared to the low‐risk group were identified. Several significantly up‐regulated gene sets were related to FGFR signaling. Further, we found up‐regulated gene sets associated with PI3K cascade, PD1 signaling, and TCR signaling. Most of the down‐regulated gene sets were associated with the regulation of cell cycle, apoptosis, and DNA damage response.

**Figure 4:**
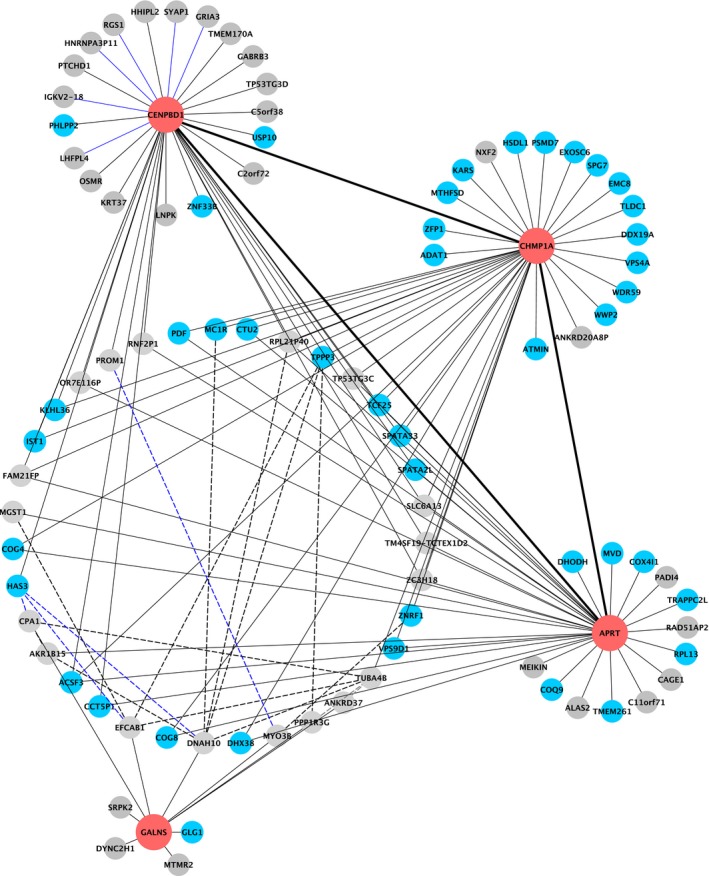
First neighborhood of the four signature genes from the reconstructed GAN in the TCGA HNSCC cohort. First neighborhood network of the four signature genes (red) extracted from the GAN. *De novo* network reconstruction was based on partial correlation of differentially expressed genes between high‐risk and low‐risk group patients in the TCGA HNSCC cohort (*n* = 98) using the GeneNet method. Black lines (edges) represent positive, and blue lines negative correlations (interactions) between genes. Dotted lines indicate the connection between the first neighborhood genes of GALNS and the first neighborhood genes of the other three signature genes. Genes, apart from the four signature genes, localized on 16q are colored in cyan.

In addition, a GAN was reconstructed. The first neighborhood (direct neighbors, *n* = 92) and second neighborhood (neighbors of first neighbors, *n* = 2972) of the four signature genes were extracted, including the information on the correlation of genes (Tables S7 and S8). Interestingly, direct network connections for three (APRT, CENPBD1, CHMP1A) of the four signature genes were observed, whereas an indirect connection was detected for galactosamine (N‐acetyl)‐6‐sulfatase (GALNS) (Fig. 4). All 50% of first neighborhood genes are localized on chromosome 16q, and a significant overrepresentation of 16q genes was observed within the first neighborhood compared to the entire network (Fisher's exact test *P* < 0.001).

Subsequent pathway enrichment analysis of genes from the first and second neighborhoods of the four signature genes revealed 493 significantly enriched pathways (FDR < 0.05) (Table S9). The top 50 identified pathways ordered according to the smallest FDR included mainly pathways associated with cell cycle, apoptosis, cell adhesion, immune response, JAK‐STAT‐signaling, signaling by SCF‐KIT, EGFR, ERBB, WNT, mTOR, and PIP3/AKT signaling.

Integration of copy number data and transcriptome data revealed a significant association (FDR < 0.1) of the genomic copy number status and the corresponding mRNA expression for 2952 out of the 7755 genes preselected for GAN reconstruction, including 64 out of 92 first neighborhood signature genes.

This represents a significant enrichment of genes within the first neighborhood with a significant association of the DNA status and their mRNA expression compared to the preselection (Fisher's exact test *P* < 0.001). Out of the 64 genes, 46 are located on chromosome 16q and thereby show a significant overrepresentation of 16q genes with significant copy‐number‐mRNA association compared to the preselection (Table S10).

## 4 Discussion

Gains of chromosomal band 16q24.3 were repeatedly shown to be associated with impaired clinical outcome of HNSCC patients after radiotherapy (Bauer *et al*., 2008; Hess *et al*., 2017). Here, we investigated the impact of 16q24.3 on mRNA expression level with regard to patients’ outcome following radio(chemo)therapy. We succeeded in the discovery and validation of a prognostic four‐gene classifier, consisting of the genes APRT, CENPBD1, CHMP1A, and GALNS located on 16q24.3. In our opinion, the fact that the signature works in both cohorts (TCGA and LMU‐KKG cohort) although exhibiting significant demographic differences underlines its robustness, which is one of the most important features of clinically applicable biomarkers.

Up to now, HPV status is the most valid and robust prognostic marker in HNSCC, whereas HPV‐positive tumors are now widely regarded as a distinct clinical entity with a different molecular pathogenesis (O'Sullivan *et al*., 2016). This emphasizes the importance for excluding possible confounding effects of HPV status and of other clinical and pathological parameters in prognostic marker research. For this reason, the independence from other clinical parameters is a particular strength of the presented classifier and allows statistically significant stratification of patient risk groups in the subgroup of HPV‐negative HNSCC.

Smoking and alcohol abuse are established risk factors for HNSCC, also contributing to a high prevalence of multiple comorbidities (e.g., cardiovascular, pulmonary, or hepatic diseases), which have a particularly high impact on the OS of HNSCC patients (Datema *et al*., 2010). Consideration of tumor‐specific clinical endpoints provides a more solid basis in prognostic marker development for HNSCC. In this sense, the four‐gene classifier was also prognostic for locoregional recurrence‐free survival and recurrence‐free survival of HNSCC patients following adjuvant radio(chemo)therapy substantiating its clinical value. These results correspond to the previously reported association of DNA gains of 16q24.3 with locoregional recurrence‐free survival in HNSCC (Bauer *et al*., 2008). The prognostic value of the signature was additionally underlined by the significant correlation of DNA gains 16q24.3 with elevated mRNA expression levels of the four classifier genes. This is important to note because copy number alterations not necessarily lead to aberrant mRNA expression of genes (Gollin, 2014; Jarvinen *et al*., 2006).

An association of genomic gains on 16q24.3 with increased mRNA expression levels was previously also observed for the DNA repair‐related gene FancA, located within this chromosomal band. Further, silencing of FancA expression in HNSCC cell lines with genomic gains on 16q24.3 resulted in significantly impaired clonogenic survival upon irradiation, whereas overexpression of FancA conferred increased survival (Hess *et al*., 2017). Interestingly, the FancA gene was not included in the best performing model when developing a low‐complexity prognostic mRNA expression signature including 16q24.3 genes. In this regard, we have indications from another project that specific transcript isoforms of FancA are associated with HNSCC patient prognosis.

The potential exploitation of the four‐gene signature as a novel therapeutic target is dependent on their biological functions and involved pathways. A first insight into the possible functional role of the signature genes was gained from the published literature, but only little information on the genes in context with HNSCC or even with cancer was available. CHMP1A (charged multivesicular body protein 1A) is known to act as a tumor suppressor in pancreatic (Li *et al*., 2008, 2009) and renal cancer (You *et al*., 2012) by inhibition of tumor cell proliferation. However, an up‐regulation of CHMP1A in HNSCC might also have a converse effect as multifunctional roles were shown for several tumor‐associated genes (Radin and Patel, 2017). Furthermore, CENPBD1 (CENPB DNA‐binding domain containing 1) plays a role in centromere formation and could, therefore, also have an influence on the cell cycle. The enzyme APRT (adenine phosphoribosyltransferase) is involved in purine metabolism (Kamatani *et al*., 1984), and deficiency of the lysosomal exohydrolase GALNS is associated with the autosomal recessive disorder Mucopolysaccharidosis IV A (Horwitz and Dorfman, 1978; Matalon *et al*., 1974). So far, APRT and GALNS have not been described in connection with cancer or cancer‐associated molecular pathways. However, a GSEA and the investigation of our reconstructed GAN revealed evidence for specific involvement of the four signature genes in pathways associated with poor clinical outcome and therapy resistance in HNSCC: for example, the EGFR/PIP3K/AKT/mTOR pathway, involved in numerous cancer‐related processes including cell cycle progression and apoptosis, is frequently altered in HNSCC and linked to therapeutic failure (Freudlsperger *et al*., 2011; Niehr *et al*., 2018). The mTOR pathway leads to the activation of the DNA‐repair machinery, thereby inducing higher radioresistance of HNSCC cells (Bose *et al*., 2013). Also the identified aberrant DNA damage response could indicate the involvement of the four signature genes in the radiation resistance of tumor cells (Hosoya and Miyagawa, 2014). Moreover, cell adhesion molecules like integrins are promising targets to overcome therapeutic resistance of HNSCC cells, especially in combination with radiation and simultaneous targeting of EGFR (Eke *et al*., 2015). Further identified pathways included the JAK‐STAT signaling, regulating cell proliferation, cell survival, and angiogenesis (Bose *et al*., 2013). In combination with aberrant Interleukin and GM‐CSF signaling, this causes markedly immune system evasion of tumors, limiting the efficacy of conventional therapies (Bose *et al*., 2013; Wu *et al*., 2015). Also the reactivation of developmental processes initiated by stem cell factor (SCF)/KIT, and Wnt signaling may play a role in the therapeutic response of HNSCC tumor cells (Ischenko *et al*., 2008). Taken together, the involvement of the four signature genes in those pathways could explain the worse clinical outcome of high‐risk patients as defined by the four‐gene classifier. Whether the functionality of the signature is related to a radiation‐resistant phenotype, which is partly suggested by the signaling pathways discussed above, or rather to tumor aggressiveness, must be investigated in future *in vitro* studies.

Moreover, since copy number alterations not necessarily lead to aberrant mRNA expression of genes (Gollin, 2014; Jarvinen *et al*., 2006), it was an important finding for us that in the case of 16q, where the signature genes are located, the gene expression levels are significantly associated with the corresponding DNA copy number status. It underlines the prognostic value of the signature based on the previous study on a DNA gain of 16q24.3 (Bauer *et al*., 2008; Hess *et al*., 2017) and its association with compromised therapy response. Furthermore, these results in combination with the analysis of the reconstructed GAN indicate a possible cluster of co‐expressed genes on chromosome 16q, including the four signature genes, with an impact on cancer‐related processes in HNSCC. Such clusters of co‐expressed genes were previously described in the published literature (Caron *et al*., 2001) and are known to consist of genes involved in the same functional pathways (Lee and Sonnhammer, 2003). Based on these facts, the cluster of co‐expressed genes on 16q could be involved in poor clinical outcome of HNSCC.

## 5 Conclusion

In conclusion, our findings do not only provide a prognostic tool for stratification of HNSCC patients treated with adjuvant radio(chemo)therapy into groups of favorable and poor prognosis independent of other clinical parameters. They also might help identifying targets for molecular therapies, since the four‐gene signature seems to be part of a functional gene expression cluster involved in HNSCC‐associated pathways.

## Author contributions

JH and KU designed the study. JH and LW designed the experiments. LW carried out the experiments. KU, MS, LW, and JH performed analysis and interpretation of the data. CM, LS, AW, CW, PB, TK, CB, and UG established the LMU‐KKG cohort including collection of clinical data and samples. JH, LW, KU, MS, and HZ interpreted the results and drafted/revised the manuscript. All authors provided critical feedback, reviewed the manuscript, and approved the final version.

## Supporting information


**Fig. S1.** Schematic workflow for the development of the classifier and the reconstruction of the gene association network with subsequent analyses.

**Fig. S2.** Kaplan–Meier curves for the endpoint overall survival for the pooled HPV‐negative patients of the radio(chemo)therapy‐treated TCGA training and validation set stratified according to the four‐gene classifier.

**Fig. S3.** DNA gains of chromosomal band 16q24.3 are associated with increased mRNA expression levels of the signature genes in radio(chemo)therapy‐treated patients of the TCGA HNSCC cohort.

**Table S1.** REporting recommendations for tumour MARKer prognostic studies (REMARK) according to McShane *et al*. (2005).

**Table S2.** Patient subset of 99 radio(chemo)therapy‐treated HNSCC patients of the TCGA cohort.

**Table S3.** Differences of demographic and histopathological parameters between the TCGA cohort and the LMU‐KKG cohort.

**Table S4.** Univariate Cox regression analyses of clinicopathological parameters and the four‐gene classifier with overall survival in the TCGA HNSCC training and validation set and the LMU‐KKG HNSCC cohort.

**Table S5.** Gene set enrichment analysis (GSEA): Significantly (FDR < 0.05) up‐regulated sets of genes between the four‐gene signature high and low‐risk groups of the TCGA cohort.

**Table S6.** Gene set enrichment analysis (GSEA): Significantly (FDR < 0.05) down‐regulated sets of genes between the four‐gene signature high and low‐risk groups of the TCGA cohort.

**Table S7.** Correlation of the first neighborhood genes of the reconstructed gene association network.

**Table S8.** Correlation of the second neighborhood genes of the reconstructed gene association network.

**Table S9.** Significantly enriched pathways (FDR < 0.05) of first and second neighborhood genes of the four signature genes from the reconstructed GAN.

**Table S10.** Significant results of the sigaR cis‐test.
