# Supplementary material for: A prognostic mRNA expression signature of four 16q24.3 genes in radio(chemo)therapy‐treated head and neck squamous cell carcinoma (HNSCC)
Source: Mol Oncol. 2018 Oct 26;12(12):2085–101. doi: 10.1002/1878-0261.12388 (PMC6275282; doi:10.1002/1878-0261.12388)
Supplement: Supplementary file 1 — Fig. S1. Schematic workflow for the development of the classifier and the reconstruction of the gene association network with subsequent analyses. [file MOL2-12-2085-s001.pdf]

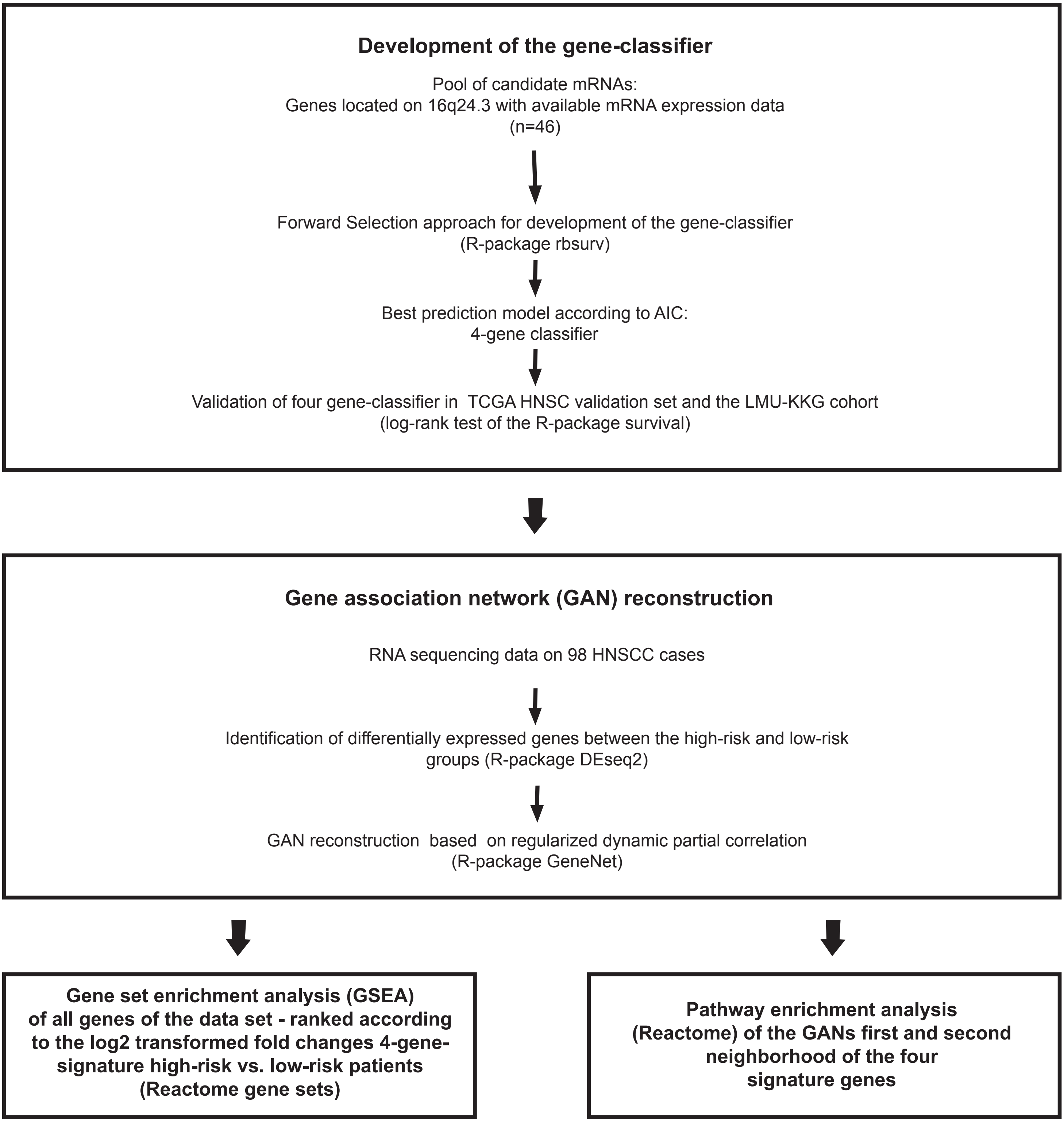

SI Fig. 1: Schematic workflow for the developement of the classifier and the reconstruction of the gene association network with subsequent analyses
A prognostic gene classifier regarding overall survival (OS) was built by applying a robust likelihood-based survival modelling approach on mRNA gene expression data (z-scores) of the TCGA training set. A stepwise forward-selection algorithm computed the partial likelihood of the Cox-proportional hazards regression model for a sequential selection of mRNAs. Best performing model was chosen based on the Akaike Information Criterion (AIC). The gene-classifier was validated in the TCGA validation set and the LMU-KKG cohort. GAN was reconstructed based on regularized dynamic partial correlation of differentially expressed genes between high-risk and low-risk patients. The functional impact of genes differentially expressed in high- and low-risk patients wasanalyzed by Gene Set Enrichment Analysis (GSEA, Reactome gene sets) and that of the first and second signature gene neighbourhoods by Reactome pathway enrichment analysis.
