# Supplementary material for: A prognostic mRNA expression signature of four 16q24.3 genes in radio(chemo)therapy‐treated head and neck squamous cell carcinoma (HNSCC)
Source: Mol Oncol. 2018 Oct 26;12(12):2085–101. doi: 10.1002/1878-0261.12388 (PMC6275282; doi:10.1002/1878-0261.12388)
Supplement: Supplementary file 2 — Fig. S2. Kaplan–Meier curves for the endpoint overall survival for the pooled HPV‐negative patients of the radio(chemo)therapy‐treated TCGA training and validation set stratified according to the four‐gene classifier. [file MOL2-12-2085-s002.pdf]

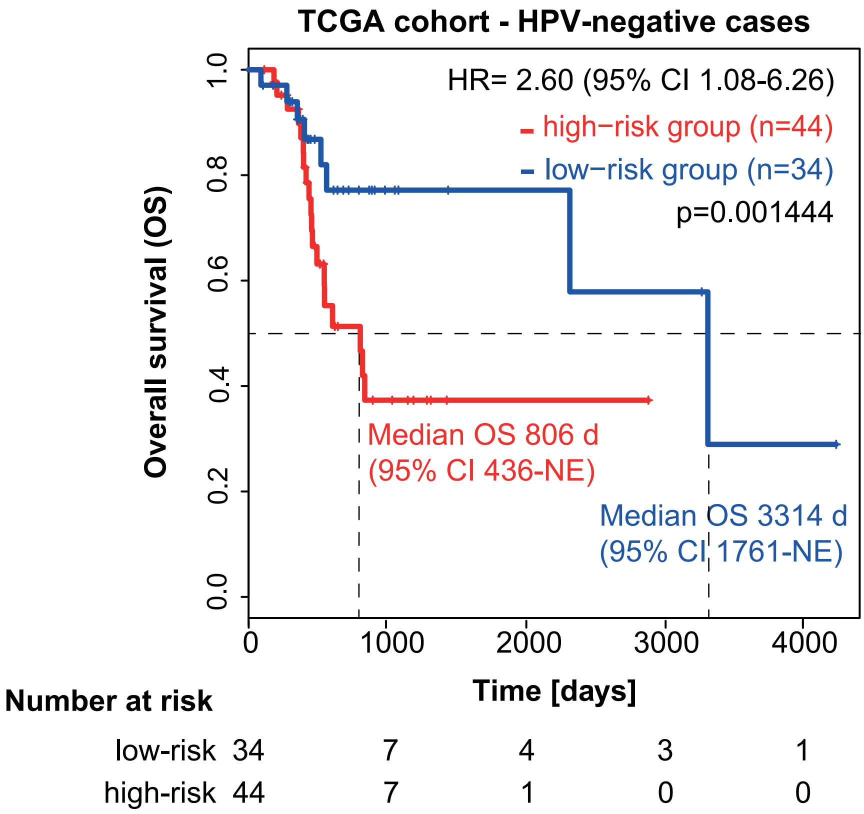

SI Fig. 2: Kaplan-Meier curves for the endpoint overall survival for the pooled HPV-negative patients of the radio(chemo)therapy-treated TCGA training and validation set stratified according to the four-gene-classifier.

Survival curves are depicted for patients of the high-risk (red, n=44) and low-risk group (blue, n=34). P-value, median overall survival times and hazard ratio (HR) with 95% contingency interval were obtained by Log-rank test and are indicated. The results are in whole based upon data generated by the TCGA Research Network: http://cancergenome.nih.gov (Cancer Genome Atlas, 2015; Cerami et al., 2012; Gao et al., 2013).

NE: not estimable
