# Supplementary material for: A prognostic mRNA expression signature of four 16q24.3 genes in radio(chemo)therapy‐treated head and neck squamous cell carcinoma (HNSCC)
Source: Mol Oncol. 2018 Oct 26;12(12):2085–101. doi: 10.1002/1878-0261.12388 (PMC6275282; doi:10.1002/1878-0261.12388)
Supplement: Supplementary file 3 — Fig. S3. DNA gains of chromosomal band 16q24.3 are associated with increased mRNA expression levels of the signature genes in radio(chemo)therapy‐treated patients of the TCGA HNSCC cohort. [file MOL2-12-2085-s003.pdf]

TCGA cohort

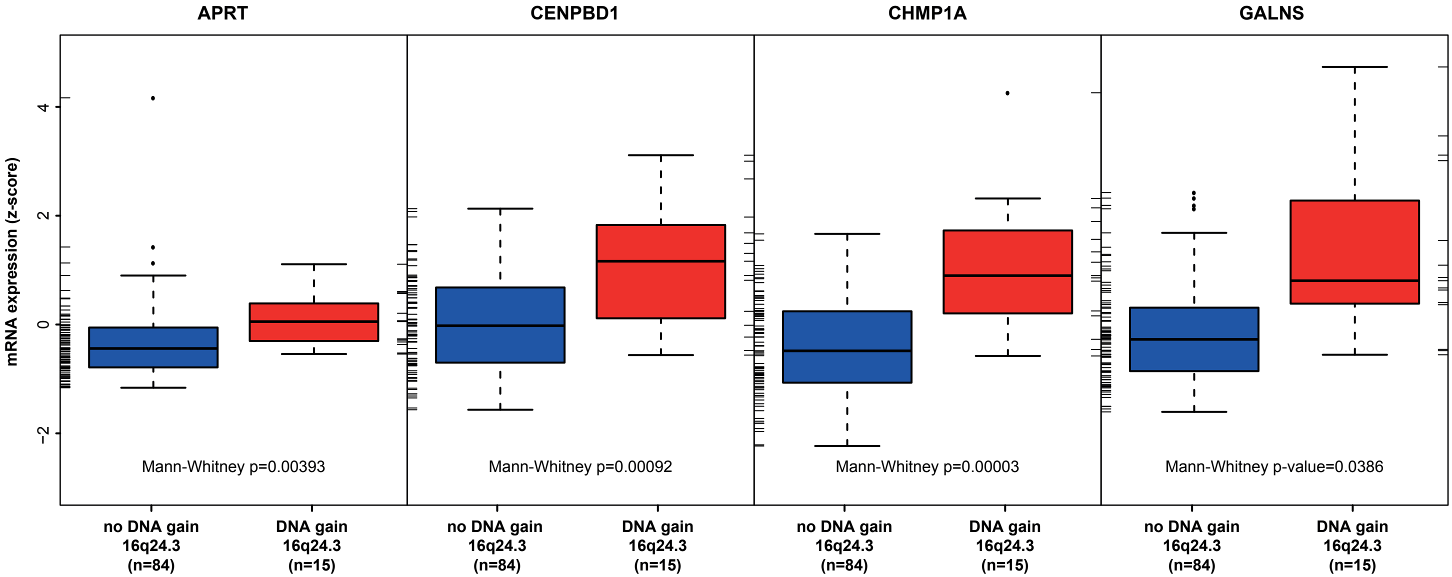

SI Fig. 3: DNA gains of chromosomal band 16q24.3 are associated with increased mRNA expression levels of the signature genes in radio(chemo)therapy-treated patients of the TCGA HNSCC cohort.
Signature genes mRNA expression levels in radio(chemo)therapy-treated HNSCC cases of the TCGA HNSCC cohort with or without DNA gain of 16q24.3. Patients with a DNA gain of 16q24.3 had statistically significantly higher mRNA expression of the signature genes compared to patients without a DNA gain on 16q24.3 in both patient sets (Mann-Whitney U test).
